# Online on-demand delivery services of food and alcohol: A scoping review of public health impacts

**DOI:** 10.1016/j.ssmph.2023.101349

**Published:** 2023-01-24

**Authors:** Cassian Duthie, Tessa Pocock, Angela Curl, Elinor Clark, Dru Norriss, Susan Bidwell, Christina McKerchar, Rose Crossin

**Affiliations:** a University of Otago Medical School, Christchurch, New Zealand; b School of Nursing, Faculty of Medical and Health Sciences, The University of Auckland, Auckland, New Zealand; c Department of Population Health, University of Otago Christchurch, Christchurch, New Zealand

**Keywords:** Alcohol delivery, Alcohol environment, Food delivery, Food environment, Meal delivery apps, Rapid delivery

## Abstract

The increase in availability of online on-demand food and alcohol delivery services has changed the way unhealthy commodities are accessed and understood. We conducted a systematic scoping review of academic and grey literature to map the current knowledge of public health and regulatory/policy outcomes arising from on-demand food and alcohol delivery (defined as delivery within 2 h). We systematically searched three electronic databases and completed supplementary forward citation searches and Google Scholar searches. In total, we screened 761 records (de-duplicated) and synthesised findings from 40 studies by commodity types (on-demand food or alcohol) and outcome focus (outlet, consumer, environmental, labour). Outlet-focused outcomes were most common (n = 16 studies), followed by consumer (n = 11), environmental (n = 7), and labour-focused (n = 6) outcomes. Despite geographical and methodological diversity of studies, results indicate that on-demand delivery services market unhealthy and discretionary foods, with disadvantaged communities having reduced access to healthy commodities. Services that deliver alcohol on-demand can also subvert current alcohol access restrictions, particularly through poor age verification processes. Underpinning these public health impacts is the multi-layered nature of on-demand services and context of the COVID-19 pandemic, which creates ongoing complications as to how populations access food and alcohol. Changing access to unhealthy commodities is an emerging issue in public health. Our scoping review considers priority areas for future research to better inform policy decisions. Current regulation of food and alcohol may not appropriately cover emerging on-demand technologies, necessitating a review of policy.

## Highlights

• On-demand food and alcohol access is growing rapidly through online apps.
• Access to unhealthy and discretionary foods may be socio-economically patterned.
• On-demand alcohol delivery may increase alcohol-related harm.
• On-demand access is complex and multi-layered, requiring a multidisciplinary focus.
• Regulation and policy may need review to consider these technological advancements.

## 1 Introduction

Consumption of unhealthy commodities, including ultra-processed foods and alcohol, are leading risk factors for chronic non-communicable diseases (Lencucha & Thow, 2019), with access being a key determinant of consumption (Swinburn et al., 2011). Increased access to unhealthy food and alcohol contributes to unhealthy environments and is associated with multiple poor public health, health, and social outcomes, including obesity (Cobb et al., 2015), unhealthy eating (Mhurchu et al., 2013), crime (Hobbs et al., 2020), psychological distress (Hobbs et al., 2021), and binge drinking (Connor et al., 2011). Furthermore, access to unhealthy commodities is a driver of the global syndemic of obesity, under-nutrition, and climate change, which affects populations globally (Swinburn et al., 2019). Controlling access to these commodities (either geographically or to specific age groups) is a regulatory lever available when aiming to build healthier environments.

In the past decade, access to food and alcohol has changed due to the growth of ‘on-demand’ delivery services, facilitated by online apps and GPS technologies. On-demand services typically coordinate between consumers, multiple outlets, and third-party delivery services, providing a single point of contact, payment, and delivery for consumers. These services utilise short delivery times, as opposed to food or alcohol ordered online for later delivery, usually on a different date. On-demand food delivery use has been steadily increasing internationally, fuelled in part by busier lifestyles and rising discretionary income (IBISWorld, 2020). On-demand food delivery services increase geographical access to food (Keeble et al., 2021a), typically market unhealthy foods (Jia et al., 2021), and may hinder the achievement of the sustainable development goals (Li et al., 2020).

Following the proliferation of on-demand food delivery, alcohol vendors have also entered this market, offering rapid alcohol delivery for immediate consumption; although, this expansion remains relatively novel compared with on-demand food. International evidence from internet alcohol vendors suggests that compliance with alcohol regulations, such as age verification or not delivering to a person who is intoxicated, may be compromised by on-demand delivery, potentially increasing underage access (Colbert et al., 2021; Fletcher et al., 2000; Van Hoof et al., 2014, 2015; Williams & Ribisl, 2012; Williams & Schmidt, 2014). Furthermore, these services utilise multiple promotions, including discounting and use of buy-now pay-later schemes, which may increase risky drinking (Colbert et al., 2020).

The rapid rise in on-demand delivery of food and alcohol also has regulatory and policy implications, as existing frameworks may have been developed based on geographical access to unhealthy commodities (Fraser et al., 2010). The geographical limits may need to be reviewed in light of on-demand delivery services offering delivery beyond the area in which individuals would be willing to travel. For example, local planning laws or liquor licensing decisions may seek to limit the geographical availability of unhealthy food or alcohol (Keeble et al., 2021c), which can then be circumvented by on-demand delivery services. Outdated regulatory frameworks allow these services to operate within a legal and policy ‘grey-zone’ (Bates et al., 2020). This ‘grey-zone’ may be exacerbated by COVID-19 lockdowns, as online services become more prominent while physical outlets close (Bates et al., 2020).

Scoping reviews differ from systematic reviews and are useful in exploring broad research questions and allowing the analysis of current evidence, including main concepts, theories, and knowledge gaps, through a systematic approach to mapping literature (Munn et al., 2018; Tricco et al., 2018). In response to this emerging public health issue, we conducted a scoping review to map and summarise current evidence on:

1) Where and how is on-demand access to food and alcohol studied?
2) What is known about the broad health impacts of on-demand food and alcohol access, at an individual or population level?
3) What are the public health regulatory and policy implications of the changing access to food and alcohol?

These questions are topical given the relative novelty and ongoing growth in on-demand delivery. We analysed on-demand delivery of both food and alcohol as many companies market the two commodities together, offering discounts when purchased in conjunction. Furthermore, it is known that the two commodities are used together, and one commodity may predict use of the other (Mojica-Perez et al., 2019).

## 2 Methods

This scoping review was conducted according to the Preferred Reporting Items for Systematic Reviews and Meta-Analyses – Extension for Scoping Reviews (PRISMA-ScR) (Tricco et al., 2018) (checklist: Supplementary Table 1). The review protocol was not prospectively registered. As the intent of this review was to scope the breadth of research, rather than reach specific conclusions, we chose not to undertake the optional risk of bias assessment (Peters et al., 2015).

### 2.1 Eligibility criteria

This scoping review included all literature from 2010 onwards, covering the period of proliferation of on-demand delivery (Curry, 2020). Full eligibility criteria are described in Table 1. There were no geographical or publication type limitations applied, although articles that did not provide original data and were not available in English language were excluded. Our criteria for ‘online’ and ‘on-demand’ food and alcohol access encompassed services that offered delivery of the commodity within 2 h via an app or website. This cut-off was defined by the authors in the absence of a standard definition and was informed by the work of Mojica-Perez et al. (2019) who use the 2 h definition to indicate on-demand delivery. This cut-off is intended to distinguish ‘on-demand’ delivery from items which typically have a longer lead time between order and delivery, even if ordered online (such as groceries, food boxes or wine box orders). However, where these products are available for delivery within 2 h, as is now sometimes the case for groceries, they would be included. If the study was unclear on whether delivery was ‘on-demand’, we excluded the study, and only included those where it was clearly stated that the study was related to on-demand delivery. In order to map and summarise current evidence related to broad health, public health, regulatory, and policy implications of on-demand delivery, we took an intentionally broad definition of health outcomes and did not limit our review to specific outcomes.

**Table 1:** Description of pre-determined eligibility criteria.

Eligibility criteria
• Published from 2010 onwards
• On-demand or fast delivery (defined as being within 2 h), either directly from or through a 3rd party application (app)
• Health or public health^a^ outcomes (to consumers or population level) and/or regulatory or policy outcomes (from a health perspective)
• Original data reported
• English language

a Broad definition of public health applied, covering labour and environmental outcomes.

### 2.2 Search strategy

Our search strategy (Supplementary Table 2) was developed in conjunction with a senior librarian, and refined through discussion with the project team, before being executed. The search strategy broadly covered on-demand access to two commodities: food and alcohol. As the growth of on-demand delivery is relatively recent, the terminology used to describe it is inconsistent and varied, hence the large number of search terms used (thirteen total; seven representing on-demand food, six representing on-demand alcohol). The search strategy included title and abstract terms and was executed on 6–7 March 2022. The search was conducted in two major databases specialising in health sciences, PubMed (1960+) and EMBASE (OVID interface, 1947+), plus one multidisciplinary database, Scopus (Elsevier). Final search results were exported into EndNote and duplicates removed.

To supplement the database search and ensure we captured the breadth of literature, two strategies were employed. First, three diverse studies (in terms of methodology, geographic setting, and commodity type) from the list of identified studies (Colbert et al., 2020; Keeble et al., 2020; Poelman et al., 2020) underwent forwards citation searches in Google Scholar. Second, grey literature was identified through Google Scholar using the search terms “online food delivery” and “online alcohol” (results sorted by relevance; limited to 2010 +). The first 800 results in both the food and alcohol search return lists were screened, as saturation has been identified at page 80 (Haddaway et al., 2015). Due to the supplementary nature of this step, the number of Google Scholar results screened, and the functional limitations of the Google Scholar database, only potentially relevant results (determined by title) were exported to EndNote. Duplicates were removed.

### 2.3 Study screening, selection, and charting

**Fig. 1:**
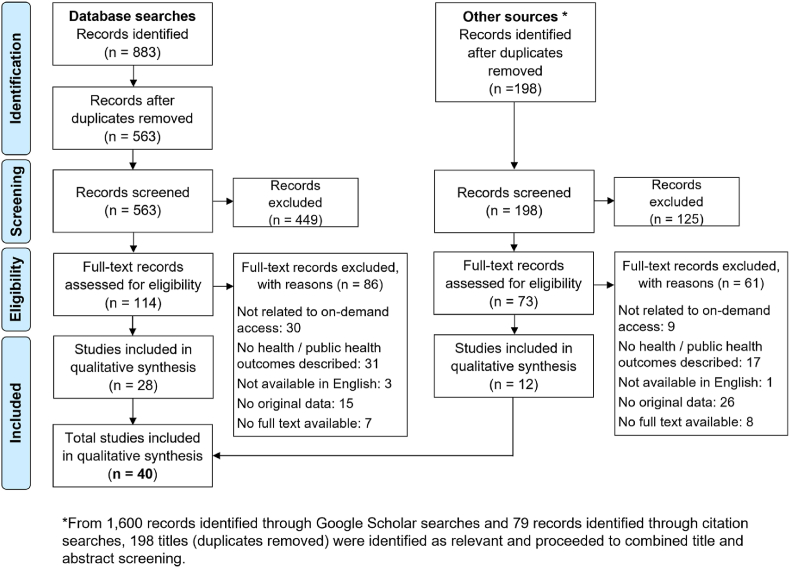
Modified PRISMA flow chart detailing the identification, screening, exclusion, and inclusion of articles.

All identified results from databases and Google Scholar were screened against the eligibility criteria. Title and abstract screening were completed first, followed by full text screening. One author completed all screening (title/abstract, full text), while a second author completed an independent 50% validation check of the title and abstract screening, and independently conducted full text screening (100%). Discrepancies were resolved through discussion. Data extraction was completed using a project-specific charting table (Supplementary Table 3). All included articles underwent independent data extraction by two authors. The extracted data was aggregated, checked for consistency, and discrepancies resolved through discussion. Patterns within the data were identified and summarised descriptively and narratively in relation to our research questions (Evans, 2002). Given the nature and extent of our database and Google Scholar searches, our search and screening processes are shown in a modified PRISMA flowchart (Fig. 1).

## 3 Results

Identification, screening, and exclusion reasons are described in Fig. 1. We screened 761 records (de-duplicated) and included 40 studies (33 journal articles, 3 pre-print articles, 2 theses, 1 conference paper, 1 report). Study characteristics, commodity type, methods, outcomes, main findings, and limitations are summarised in Supplementary material 4.

### 3.1 Where and how is on-demand access to food and alcohol studied?

Thirty-three studies (83%) explored on-demand ‘food’ delivery, six (15%) explored on-demand ‘alcohol’ delivery, and one (3%) explored a combination of on-demand ‘food’ and ‘alcohol’ delivery. There was geographical breadth in research of on-demand delivery, covering 15 countries: Australia (n = 11), China (n = 10), United States of America (USA) (n = 7), United Kingdom (UK) (n = 6), New Zealand (n = 4), Brazil (n = 3), Canada (n = 3), Netherlands (n = 3), Indonesia (n = 1), Ireland (n = 1), Japan (n = 1), Malaysia (n = 1), Mexico (n = 1), Scotland (n = 1), and Sweden (n = 1). Five studies incorporated multiple geographical areas (e.g., compared between cities), ranging from two (Partridge et al., 2020) to six areas (Colbert et al., 2021). Publication dates ranged from 2015 to 2022; 12 studies were published in 2020, 20 in 2021, and five by March 2022.

#### 3.1.1 Study methods

The study methods for on-demand ‘food’ delivery research were broad, comprising 12 different approaches (see Supplementary Table 4 for full details). The most common methods were surveys (n = 13; 32%), sampling of apps and/or websites (n = 11; 27%), interviews (n = 5; 12%), and life-cycle assessment (n = 4; 10%). Multiple methods were employed in seven studies (Arunan & Crawford, 2021; Babar et al., 2021; Björkbom & Nguyen, 2021; Hodges Jr, 2020; Maimaiti et al., 2020; X. Wang, Xu, et al., 2021; Zhang et al., 2022). For example, Arunan and Crawford (2021) used a combination of consumer surveys, restaurant manager interviews, life cycle assessment, and food packaging assessment in their study. For on-demand ‘alcohol’ delivery, the reported methods were survey (n = 2; 33%), website content analysis (n = 1; 17%), policy review (n = 1; 17%), audit of regulatory controls (n = 1; 17%), and mystery shopping studies (n = 1; 17%). For the one article which explored on-demand ‘food’ and ‘alcohol’ delivery, desktop audits of apps and websites were conducted. Units of study included consumers (customers), outlets, employees of services, packaging samples, advertisements, menu items, completed food deliveries, and policies.

Sample sizes were typically large, ranging from 35 to 3067 consumers (n = 14 studies), 65 to 29,232 outlets (n = 12), 25 to 883 employees (n = 6), 5 to 810 food packaging/waste samples (n = 4), 581 to 1754 advertisements (n = 3), 759 to 13,841 menu items (n = 3), and 40,941 to 4.8 billion data points on completed food deliveries (n = 3 studies). One study also reviewed policies covering 77 jurisdictions.

#### 3.1.2 Study outcomes

**Fig. 2:**
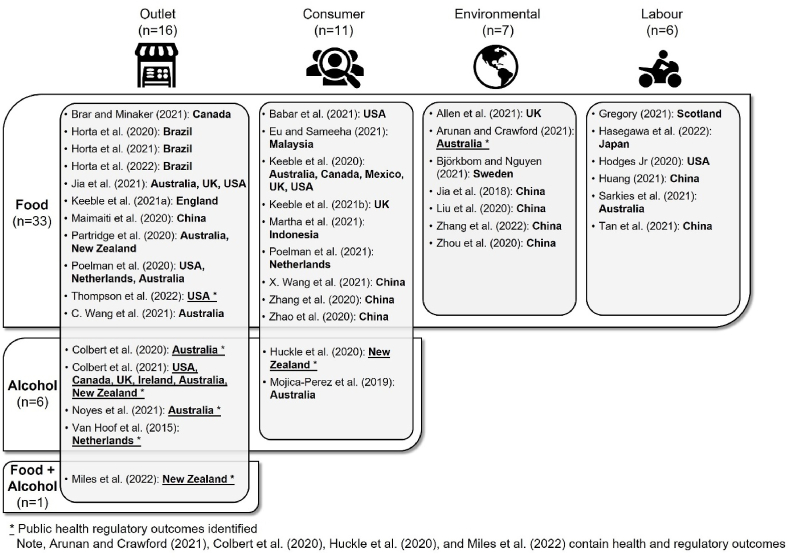
Categorisation matrix demonstrating the studies conducted across commodity types (food or alcohol) and focus (outlet, consumer, environmental, labour).

There were a wide variety of health-related outcomes and public health regulatory and policy implications described in the identified studies. To synthesise these outcomes, we created a matrix (Fig. 2) and categorised outcomes according to commodity type (on-demand food or alcohol) and the focus of the outcomes (outlet-focused, consumer-focused, environmental-focused, labour-focused). The categorisation based on outcome focus emerged from the review. Fig. 2 also demonstrates the eight studies which were identified as containing public health regulatory outcomes. In several circumstances, the on-demand services theoretically delivered both food and alcohol; yet the studies did not specify whether alcohol was delivered alongside food. In these circumstances, studies were summarised under the commodity type of 'food’ (Allen et al., 2021; Björkbom & Nguyen, 2021; Hasegawa et al., 2022; Sarkies et al., 2021).

### 3.2 What is known about the broad health impacts of on-demand food and alcohol access, at an individual or population level?

Outlet-focused outcomes (n = 16 studies) were predominant across commodity types of ‘food’ and ‘alcohol’, including for the study which combined on-demand food and alcohol delivery (Fig. 2, Supplementary Table 4). Outlet-focused outcomes included geographical food access, healthfulness (food-related studies), alcohol policies, safeguards, and access (alcohol-related studies). Consumer-focused outcomes (n = 11 studies) were the next most common across commodity types. Consumer-focused outcomes included dietary diversity, self-reported body mass index (BMI), health risks (food-related studies), and alcohol use patterns (alcohol-related studies). Environmental (n = 7 studies) and labour-focused (n = 6 studies) outcomes were only present in food-related studies. Environmental-focused outcomes covered greenhouse gas (GHG) emissions, energy consumption, and waste generation, while labour-focused outcomes included worker risks and injury, income, and labour conditions. Of the studies that considered consumers or employees of on-demand services, the majority included participants aged over 18 years (when ages were stated), with one exception that included children (X. Wang, Xu, et al., 2021).

#### 3.2.1 Outlet-focused ‘food’ studies

When analysing outlet-focused ‘food’ research (n = 11 studies), ten studies (91%) analysed the foods sold by grouping them into ‘healthiness’-related categories. A common limitation across studies was the investigation of healthiness based on food being offered for sale, rather than what was most commonly purchased. The majority of outlets, menu items, advertisements, and discounts offered were for unhealthy or discretionary foods^1^ (Brar & Minaker, 2021; Horta et al., 2021; Horta et al., 2022; Horta et al., 2020; Jia et al., 2021; Maimaiti et al., 2020; Partridge et al., 2020; Poelman et al., 2020; Thompson et al., 2022; Wang et al., 2021). Three studies included measures of socio-economic deprivation within their analyses, concluding that advertised food types and access to on-demand services varied according to area-level deprivation, and indicating that healthier food outlets were more accessible in the least disadvantaged areas (Partridge et al., 2020; Poelman et al., 2020) but that the percentage of food outlets accepting orders online increased with deprivation (Keeble et al., 2021a). In one study considering geographical access, on-demand food delivery services substantially increased access to foods prepared away from the home (Brar & Minaker, 2021).

#### 3.2.2 Consumer-focused ‘food’ studies

Of the consumer-focused ‘food’ research (n = 9 studies), Household Dietary Diversity Scores (HDDS; reflection of household food accessibility) (Zhang et al., 2020; Zhao et al., 2020) and self-reported BMI (Babar et al., 2021; Keeble et al., 2020, 2021b) were the most common health outcomes (n = 5; 56%). HDDS was not significantly different between those who typically used on-demand food delivery, compared to those who did not (Zhao et al., 2020), while HDDS was also found to be significantly higher among those who increased consumption of on-demand food during the COVID-19 pandemic, even after adjustment for family income and location (Zhang et al., 2020). In relation to BMI, on-demand food delivery use was not different between BMI categories (Keeble et al., 2020) and had no association with number of accessible online food outlets (Keeble et al., 2021b). Conversely, one study which aggregated BMI data across 85 counties, before and after the introduction of a major on-demand food delivery service (2005–2019), found that on-demand delivery service entry was associated with a 0.5% increase in average population BMI (Babar et al., 2021).

Health outcomes across consumer-focused ‘food’ research also covered domestic cooking and in-restaurant dining behaviours (Babar et al., 2021), perceptions of healthy food (Eu & Sameeha, 2021), online food outlet access and use (Keeble et al., 2021b), consumption of high risk foods (Martha et al., 2021), use of on-demand delivery services due to COVID-19 lockdowns (Poelman et al., 2021), and health risks related to food consumption from disposable plastic containers (X. Wang, Xu, et al., 2021). Findings were inconsistent, reflective of the wide range of health outcomes considered. On-demand food delivery was found to reduce daily meal preparation time (Babar et al., 2021), while greater on-demand food access was found to substantially increase odds of reporting online food delivery use (Keeble et al., 2021b). Food choices, either healthy or unhealthy, were not associated with perceptions of healthy food availability (Eu & Sameeha, 2021), yet having high preference for using on-demand food delivery increased high-risk food consumption through such services (Martha et al., 2021). Finally, high food temperatures, high consumption frequency, and high-fat food characteristics were suggested to exacerbate the health risks related to consumption of food from disposable plastic containers (X. Wang, Xu, et al., 2021).

#### 3.2.3 Environmental-focused ‘food’ studies

Among the seven studies analysing environmental-focused health impacts of on-demand ‘food’ delivery, several key patterns were identified. First, there was wide use of delivery modes that used fossil fuels, often over short distances (2–4 km), and conflicting with what companies listed as primary transport modes (e.g., bicycle) compared to actual modes (e.g., moped, car) (Björkbom & Nguyen, 2021). Meals delivered by car or petrol moped had far greater GHG emissions and transport intensity (kerb occupancy, distance travelled) compared to bicycles (Allen et al., 2021). Second, waste from food deliveries was remarkable and increasing (Jia et al., 2018). Food packaging was a strong contributor to total municipal waste (15.7% of total) (Liu et al., 2020), while food delivery waste disposal presented the largest share of carbon emissions (75%), followed by food and waste transportation (Zhang et al., 2022). Finally, raw packaging material production was a major source of CO_2_ emissions (Arunan & Crawford, 2021; Liu et al., 2020; Zhou et al., 2020), followed by incineration (Zhou et al., 2020). In modelling three different on-demand packaging scenarios (current packaging and waste disposal patterns; paper substitution; tableware sharing), one study found that tableware sharing could reduce waste generation by up to 92%, and environmental emissions and water consumption by more than two-thirds, compared to current patterns (Zhou et al., 2020).

#### 3.2.4 Labour-focused ‘food’ studies

In relation to labour-focused health impacts (n = 6 studies), employees of on-demand ‘food’ delivery services experienced diverse risks, which were amplified by the pandemic and algorithmic labour processes (Gregory, 2021; Huang, 2021). Income precarity developed from unpredictable wages (Gregory, 2021), often below minimum wage (Hasegawa et al., 2022), and restricted access to pandemic-related benefits and support (because delivery drivers were employed as contractors) (Huang, 2021). Physical risks and harm included road incidents, COVID-19 infection, and environmental exposure (Gregory, 2021; Huang, 2021). In two studies focusing specifically on road incidents, the arrival of on-demand food delivery services to 18 Chinese cities was associated with an average increase of 611 road incidents and 51 deaths per city per year, resulting in substantial personal and economic costs (3.65 million CNY per city/year) (Tan et al., 2021). Furthermore, commercial cyclists were more likely to be struck by a motor vehicle than other cyclists (Sarkies et al., 2021). Additional risks include lack of food safety training, stress caused by pressure to maintain rating scores (Hodges Jr, 2020), and racialised environments (Huang, 2021).

#### 3.2.5 Alcohol-related outlet and consumer-focused studies

Six studies exclusively explored on-demand ‘alcohol’ delivery services, with only three of these studies incorporating health-related outcomes (the rest covered regulatory and/or policy implications; described in next section). The outlet-focused health outcome study covered alcohol purchasing costs (Colbert et al., 2020). On-demand alcohol delivery services were found to offer multiple types of alcohol at very low costs, with most of the 65 retailers investigated offering bulk buy discounts and 13.8% allowing alcohol purchases through ‘buy now, pay later’ schemes (Colbert et al., 2020). Two studies incorporated consumer-focused health outcomes, using 12-month alcohol use patterns (Mojica-Perez et al., 2019) or patterns of heavy drinking (Huckle et al., 2020) as outcome measures. Those who used on-demand alcohol delivery had 75% higher odds of heavier drinking in the past week (Huckle et al., 2020) and generally drank more heavily than those whose order took longer than 2 h (28.6% had 11+ drinks on that occasion) (Mojica-Perez et al., 2019). Furthermore, consumers in both studies were able to order alcohol online while intoxicated (Huckle et al., 2020; Mojica-Perez et al., 2019), with over a quarter stating they would have had to stop drinking alcohol if the delivery service was not available (Mojica-Perez et al., 2019). Finally, for many individuals ordering on-demand food online, their chosen food outlet was influenced by alcohol availability, thus conferring both alcohol and nutritional health impacts (Mojica-Perez et al., 2019).

#### 3.2.6 Combined ‘food’ and ‘alcohol’ outlet-focused study

The one study which incorporated ‘food’ and ‘alcohol’ outlet-focused health outcomes, explored the availability of unhealthy on-demand commodities (Miles et al., 2022). This study aligns with the findings above, indicating substantial access across urban and rural areas in New Zealand and high use of promotions (97%).

### 3.3 What are the public health regulatory and policy implications of the changing access to food and alcohol?

Eight studies (n = 2 food; n = 5 alcohol; n = 1 food and alcohol) incorporated regulatory or policy outcomes from a health perspective (Fig. 2, Supplementary Table 4). The two ‘food’ studies considered different regulatory/policy outcomes. In quantifying packaging-related GHG emissions associated with online food delivery orders, Arunan and Crawford (2021) predicted an annual increase in emissions of 132% by 2024. Thompson et al. (2022) explored compliance with California’s Healthy-By-Default Beverage law (mandating water or unflavoured milk be default drinks with children’s meals) for meals sold through online platforms from restaurants in low-income neighbourhoods and concluded that most on-demand delivery services are not offering healthy children’s beverages consistent with state law. The five ‘alcohol’ studies (Colbert et al., 2020, 2021; Huckle et al., 2020; Noyes et al., 2021; Van Hoof et al., 2015) and the one ‘food’ and ‘alcohol’ study (Miles et al., 2022) considered age compliance/verification at purchase or delivery, indicating that compliance differed substantially across geographical location and measurement type. These studies highlighted inadequate age verification processes (some services found it satisfactory if an older third-party accepted the product on behalf of underage consumers) (Colbert et al., 2020, 2021; Huckle et al., 2020; Noyes et al., 2021; Van Hoof et al., 2015), insufficient training of alcohol delivery drivers (Colbert et al., 2021), inconsistent application of liquor laws (namely, age compliance) governing on-demand settings (Noyes et al., 2021), and irregularities in the legislative language used across jurisdictions, creating uncertainty around age compliance/verification checks (Colbert et al., 2021).

Birth date entry, age warning messages/pop-ups, and policies around refusal of delivery to intoxicated persons were inconsistently observed across on-demand alcohol delivery services (Colbert et al., 2020; Miles et al., 2022; Noyes et al., 2021). One New Zealand study observed that all alcohol-only delivery services had birth date entry, age warning pop-ups, and intoxication law messages; however, for services delivering food and alcohol, pop-ups were present on only 57% of services (birth date entry and intoxication law messages remained high) (Miles et al., 2022). Similarly, differences in ordering limits placed on age-restricted items were observed, with 96% of alcohol-only services specifying a limit compared to 43% of food and alcohol services (Miles et al., 2022). In contrast to physical store requirements, few Organisation for Economic Co-operation and Development (OECD) jurisdictions required age warnings to be present on websites (Colbert et al., 2021). Furthermore, alcohol delivery from on-demand services was permitted outside maximum trading hours for licenced premises in seven OECD jurisdictions (Colbert et al., 2021).

### 3.4 What is the impact of COVID-19 on access to food and alcohol?

While investigation of COVID-19-related changes in on-demand access was not intended as an outcome from this review, reference to COVID-19 was common across both ‘food’ and ‘alcohol’-focused studies, necessitating the inclusion of this section. Ten (25%) of the included studies were set within the context of the pandemic (n = 8 food; n = 2 alcohol), and a further three acknowledged changing COVID-19 situations as a research limitation (Eu & Sameeha, 2021; Gregory, 2021; Keeble et al., 2021b). Furthermore, only three studies (8%) were published prior to the COVID-19 pandemic (i.e., 2015–2019); however, it is unknown how many studies published during pandemic times used data collected prior to the pandemic.

The ten studies set within the context of the pandemic incorporated outlet-focused, consumer-focused, and labour-focused outcomes. On-demand ‘food’ outlets tended to promote unhealthy or discretionary menu items during pandemic restrictions (Horta et al., 2021, 2022; Jia et al., 2021), with some outlets using specific COVID-19 marketing strategies (e.g., combatting the pandemic, selling social distancing) to promote discretionary items (Jia et al., 2021). Consumer use of ‘food’ delivery services during restrictions differed; service use did not substantially change among adults in the Netherlands (Poelman et al., 2021), while service use increased for a substantial number of adults in China (Zhang et al., 2020). Two studies found conflicting evidence regarding possible changes in dietary diversity related to use of on-demand food delivery during pandemic restrictions (Zhang et al., 2020; Zhao et al., 2020). Labour-focused outcomes indicated that the pandemic is impacting income and job security, as well as compounding racialised environments (e.g., delivery drivers were labelled as ‘virus carriers’) (Hasegawa et al., 2022; Huang, 2021). Finally, on-demand ‘alcohol’ delivery during COVID-19 pandemic restrictions was associated with a greater proportion of consumers purchasing from online vendors and drinking more heavily, with many being first-time users of such services (Huckle et al., 2020). Access to online alcohol delivery services was heightened during pandemic restrictions with over two-thirds (69%) of 77 jurisdictions studied relaxing restrictions for home alcohol delivery, and 13 of these making permanent changes (Colbert et al., 2021).

## 4 Discussion

The increase in availability and use of on-demand ‘food’ and ‘alcohol’ delivery represents a change to unhealthy commodity environments, impacting public health. The proliferation of on-demand delivery services coincides with the COVID-19 pandemic; however, underlying trends in health outcomes from before the COVID-19 pandemic are unclear. Through a systematic scoping review, we identified, screened, and synthesised research that studied on-demand delivery. Specifically, we categorised studies according to commodity type (food or alcohol) and the focus of the outcomes (outlet, consumer, environmental, labour) before synthesising findings around individual and public health outcomes, regulatory and policy implications, and the impact of COVID-19 on access to unhealthy commodities. Despite geographical and methodological diversity across the 40 studies, our findings indicate that (1) on-demand delivery broadens consumer access to unhealthy food and alcohol, with evidence of food access being socio-economically patterned; (2) on-demand ‘alcohol’ delivery may increase alcohol-related harm, particularly underage and binge drinking; and (3) on-demand access presents a multi-layered problem, compounded by the COVID-19 pandemic and presenting ongoing issues for food and alcohol access. We draw on these findings to present priority areas for future research and policy.

On-demand ‘food’ delivery services predominantly market unhealthy and discretionary foods (e.g., Horta et al., 2020; Partridge et al., 2020; Poelman et al., 2020), even employing specific COVID-19 marketing strategies to promote discretionary items (Jia et al., 2021). On-demand food delivery services also contribute to health inequities, with disadvantaged communities having greater access to unhealthy on-demand delivery services (Keeble et al., 2021a; Partridge et al., 2020; Poelman et al., 2020). Furthermore, such services circumvent geographical controls over discretionary food outlet location, enhancing access to unhealthy and discretionary foods (Brar & Minaker, 2021; Keeble et al., 2021a). These findings are not unexpected, considering there is a strong association between neighbourhood deprivation and fast-food availability (Fraser et al., 2010; Pearce et al., 2007). However, despite greater availability of on-demand outlets in more deprived areas, individuals with higher educations and greater incomes are more likely to use on-demand food delivery (Keeble et al., 2020), illustrating the importance of further research considering the interaction between location of outlets and consumer behaviour in relation to deprivation. Overall, the evidence base is limited, and further research should focus on the public health impacts of on demand services in order to support development of evidence based regulation. The impacts of on-demand food delivery on individual consumer health also needs to be clarified, as a wide variety of health outcomes were identified in our review, predominantly HDDS and self-reported BMI. Our findings highlight inconsistencies in how nutritional value was assessed, favouring outlet-focused analysis of menu items rather than what foods were consumed, ultimately limiting knowledge about the public health impacts of these services and recommendations for regulation. Standardised nutrition measures at the consumer-level, and assessment of the nutritional quality of food consumed, would facilitate meaningful comparison across studies and development of evidence-based recommendations. Furthermore, to address the paucity of diet or body weight-related public health regulations identified in our review, future studies should address and develop evidence-based regulations around access to on-demand services.

Current evidence suggests that on-demand ‘alcohol’ services may increase alcohol-related harm, particularly underage and binge drinking (e.g., Huckle et al., 2020; Mojica-Perez et al., 2019; Van Hoof et al., 2015). Combating harmful drinking is complex, particularly when considering health inequity impacts. The association between deprivation and binge drinking is known (Fone et al., 2013). Research has further identified an association between on-demand alcohol delivery services and binge drinking, even implicating food retailers in the picture with alcohol availability determining the use of on-demand food services (Mojica-Perez et al., 2019). Regulating the availability of alcohol is one effective approach to address harmful drinking (Popova et al., 2009); however, our findings indicate that on-demand services may directly and indirectly subvert access restrictions, particularly through poor age verification processes, no liability of third-party delivery services (Colbert et al., 2020; Huckle et al., 2020), and substantial geographical (Miles et al., 2022) and temporal access (Colbert et al., 2021). Inconsistencies and lack of regulatory control (Colbert et al., 2021; Noyes et al., 2021) governing on-demand alcohol services requires urgent policy reform (Huckle et al., 2020).

COVID-19 related changes in on-demand alcohol legislation further complicates the issue of underage access and binge drinking. There is a significant body of evidence that alcohol availability and access is a key determinant of harm, and thus an important point of intervention (Anderson et al., 2009). Temporary and permanent changes to online retailer requirements substantially changed how alcohol could be accessed during and after pandemic restrictions (Colbert et al., 2021). Furthermore, behavioural changes in on-demand alcohol purchasing and drinking habits were also observed during the pandemic (Huckle et al., 2020). In light of this, jurisdictions that currently place geographical controls on alcohol sales may want to revisit regulations to control alcohol access and minimise the potential health equity impacts. In addition, as studies related to on-demand alcohol predominantly considered age verification and regulatory requirements of online retailers, future research should involve consumers to understand impacts of on-demand alcohol delivery to both individual and population health. Increasing access to alcohol via on-demand delivery, in combination with regulatory changes and consumption patterns that occurred during the pandemic, may result in an exacerbation of alcohol-related harm. Given that on-demand food and alcohol delivery are connected (Mojica-Perez et al., 2019), it may also be beneficial to include on-demand tobacco and e-cigarette products in future research to broaden our understanding of connections between unhealthy commodities (Miles et al., 2022; Williams et al., 2015, 2017).

On-demand access presents a multi-layered problem requiring a multidisciplinary focus. Our findings demonstrated four key areas within on-demand access research to-date (outlets, consumers, environmental, labour), while also elaborating on underlying public health and regulatory patterns across the data: promotion of discretionary items; socio-economic patterning of food access; direct/indirect facilitation of underage and binge drinking; environmental impacts of food/packaging waste and transport related emissions; precarious labour market and escalating risks to workers; and specific strategies to market unhealthy commodities, including COVID-19 related promotions. Further elucidating the links between on-demand access and broad measures of public health is especially important considering the links between obesity, under-nutrition, and climate change (Swinburn et al., 2019). COVID-19 has concurrently affected how populations access food and alcohol, creating particularly potent health implications (Colbert et al., 2021; Hasegawa et al., 2022; Horta et al., 2021, 2022; Huang, 2021; Huckle et al., 2020; Jia et al., 2021). As only one-quarter of our included studies were conducted specifically within the context of the pandemic, it is unknown how behavioural changes and on-demand consumption patterns will persist. Further exploration of COVID-19 impacts on on-demand food and alcohol delivery over time are needed. In addition, as we emerge from pandemic restrictions, further consideration of different population groups (particularly, adolescent populations who were largely absent from our included articles) and ongoing monitoring of marketing strategies are needed. This last suggestion is also particularly relevant given increasing discussion around regulating digital marketing of unhealthy commodities to children (Buchanan et al., 2018; World Health Organization, 2016).

### 4.1 Strengths and limitations

A strength of our review is linking of on-demand food and alcohol delivery services, two commodities that are not typically studied together in the literature but may interact in determining consumer access (Mojica-Perez et al., 2019). A second strength is the broad range of terminology used in our search strategy, and the combination of electronic database, forwards citation, and supplementary Google Scholar searches that included grey literature, to capture a wide understanding of on-demand access.

Our study also has several limitations. First, in the absence of a standard definition of on-demand delivery, we used a definition of delivery *within 2 h* to distinguish on-demand delivery from home delivery of food boxes or wine orders. We suggest this definition should be used consistently to distinguish on-demand delivery in future research. Studies that did not explicitly include on-demand delivery within our pre-defined delivery window were excluded (n = 39; Fig. 1). Second, we only searched three electronic databases and completed supplementary grey literature searches through Google Scholar and forward citation searches. While our search strategy was designed in consultation with a senior librarian, it is possible that additional relevant studies and grey literature were not identified. Third, due to the heterogeneity of health-related outcomes, it was not always possible to directly compare study findings. Fourth, the ongoing COVID-19 pandemic creates uncertainty around the continued role on-demand services will have and the long-term impact of our recommendations. Despite these challenges, this scoping review enabled us to present a first-of-its-kind picture of how on-demand delivery has been studied to date.

## 5 Conclusion

This scoping review mapped the current knowledge of public health and regulatory/policy outcomes arising from on-demand food and alcohol delivery – an emerging public health issue. Our findings indicated that on-demand services are likely to be worsening existing health issues and inequities for consumers, due to enhanced access to unhealthy food and alcohol commodities and evidence of socio-economic patterning of on-demand food. Furthermore, on-demand access is complex and multi-layered, requiring consideration of a broad range of public health factors. The enduring impact of the COVID-19 pandemic continues to complicate how populations access food and alcohol. Collectively, our findings provide a basis for future research to better understand the public health impacts. Further research is required to inform evidence-based policy and regulatory decisions to adapt to the rapid change in on-demand food and alcohol environments.

## CRediT author statement

**Cassian Duthie**: Conceptualization, Methodology, Investigation, Validation, Data Curation, Formal analysis, Writing - Original Draft, Writing - Review & Editing, Visualization, Project administration; **Tessa Pocock**: Conceptualization, Investigation, Validation, Data Curation, Formal analysis, Writing - Original Draft, Writing - Review & Editing, Visualization, Project administration; **Angela Curl**: Conceptualization, Methodology, Validation, Formal analysis, Writing - Review & Editing, Funding acquisition; **Elinor Clark**: Validation, Formal analysis, Writing - Review & Editing; **Dru Norriss**: Formal analysis, Writing - Review & Editing; **Susan Bidwell**: Formal analysis, Writing - Review & Editing; **Christina McKerchar**: Conceptualization, Methodology, Validation, Formal analysis, Writing - Review & Editing, Funding acquisition; **Rose Crossin**: Conceptualization, Methodology, Validation, Formal analysis, Writing - Review & Editing, Supervision, Funding acquisition.

## Funding

This study was conducted with financial support from 10.13039/100008247the University of Otago (Christchurch) Division of Health Sciences for a summer studentship, plus funding from a 10.13039/100008247University of Otago Research Grant, and a Lottery Health Grant (both awarded to authors RC, AC, CM). The funders had no role in study design, conduct, or the decision to publish.

## Ethical statement

This study did not require ethical approval as it was a review.

## Declarations of competing interest

None.

## Data Availability

The data are previously published studies and these are appropriately referenced and described, for others to access.

## Footnotes

1 Foods that are not necessary for the human body’s daily nutrient requirements.
