# Supplementary material for: Online on-demand delivery services of food and alcohol: A scoping review of public health impacts
Source: SSM Popul Health. 2023 Jan 24;21:101349. doi: 10.1016/j.ssmph.2023.101349 (PMC9950721; doi:10.1016/j.ssmph.2023.101349)
Supplement: Multimedia component 1 [file mmc1.docx]

**Supplementary Table 1 – Preferred Reporting Items for Systematic reviews and Meta-Analyses extension for Scoping Reviews (PRISMA-ScR) Checklist**

| **SECTION** | **ITEM** | **PRISMA-ScR CHECKLIST ITEM** | **REPORTED ON PAGE #** |
| --- | --- | --- | --- |
| **TITLE** | | | |
| Title | 1 | Identify the report as a scoping review. | 0 – Title page |
| **ABSTRACT** | | | |
| Structured summary | 2 | Provide a structured summary that includes (as applicable): background, objectives, eligibility criteria, sources of evidence, charting methods, results, and conclusions that relate to the review questions and objectives. | 1 |
| **INTRODUCTION** | | | |
| Rationale | 3 | Describe the rationale for the review in the context of what is already known. Explain why the review questions/objectives lend themselves to a scoping review approach. | Introduction, para. 1-5 |
| Objectives | 4 | Provide an explicit statement of the questions and objectives being addressed with reference to their key elements (e.g., population or participants, concepts, and context) or other relevant key elements used to conceptualize the review questions and/or objectives. | End of introduction, para. 5-6 |
| **METHODS** | | | |
| Protocol and registration | 5 | Indicate whether a review protocol exists; state if and where it can be accessed (e.g., a Web address); and if available, provide registration information, including the registration number. | Methods section, para. 1 |
| Eligibility criteria | 6 | Specify characteristics of the sources of evidence used as eligibility criteria (e.g., years considered, language, and publication status), and provide a rationale. | Table 1; Methods section 2.1 |
| Information sources* | 7 | Describe all information sources in the search (e.g., databases with dates of coverage and contact with authors to identify additional sources), as well as the date the most recent search was executed. | Methods section 2.2 |
| Search | 8 | Present the full electronic search strategy for at least 1 database, including any limits used, such that it could be repeated. | Supplementary Table 2 |
| Selection of sources of evidence† | 9 | State the process for selecting sources of evidence (i.e., screening and eligibility) included in the scoping review. | Methods section 2.3 |
| Data charting process‡ | 10 | Describe the methods of charting data from the included sources of evidence (e.g., calibrated forms or forms that have been tested by the team before their use, and whether data charting was done independently or in duplicate) and any processes for obtaining and confirming data from investigators. | Methods section 2.3 |
| Data items | 11 | List and define all variables for which data were sought and any assumptions and simplifications made. | Supplementary Table 3 |
| Critical appraisal of individual sources of evidence§ | 12 | If done, provide a rationale for conducting a critical appraisal of included sources of evidence; describe the methods used and how this information was used in any data synthesis (if appropriate). | Not completed |
| Synthesis of results | 13 | Describe the methods of handling and summarizing the data that were charted. | Methods section 2.3 |
| **RESULTS** | | | |
| Selection of sources of evidence | 14 | Give numbers of sources of evidence screened, assessed for eligibility, and included in the review, with reasons for exclusions at each stage, ideally using a flow diagram. | Figure 1; Results, para. 1 |
| Characteristics of sources of evidence | 15 | For each source of evidence, present characteristics for which data were charted and provide the citations. | Supplementary Table 4 |
| Critical appraisal within sources of evidence | 16 | If done, present data on critical appraisal of included sources of evidence (see item 12). | Not completed |
| Results of individual sources of evidence | 17 | For each included source of evidence, present the relevant data that were charted that relate to the review questions and objectives. | Supplementary Table 4; Results section 3.1, 3.2, 3.3, 3.4 |
| Synthesis of results | 18 | Summarize and/or present the charting results as they relate to the review questions and objectives. | Results section 3.1, 3.2, 3.3, 3.4 |
| **DISCUSSION** | | | |
| Summary of evidence | 19 | Summarize the main results (including an overview of concepts, themes, and types of evidence available), link to the review questions and objectives, and consider the relevance to key groups. | Discussion section, para. 1-5 |
| Limitations | 20 | Discuss the limitations of the scoping review process. | Discussion section 4.1 |
| Conclusions | 21 | Provide a general interpretation of the results with respect to the review questions and objectives, as well as potential implications and/or next steps. | Conclusion, para. 1 |
| **FUNDING** | | | |
| Funding | 22 | Describe sources of funding for the included sources of evidence, as well as sources of funding for the scoping review. Describe the role of the funders of the scoping review. | Acknowledged |

**Supplementary Table 2 – Search strategies and number of results obtained across the three searched databases**

| **PubMed (1960+)** | |  |
| --- | --- | --- |
| # | Search Syntax | 6/03/2022 |
| Food |  |  |
| 1 | "Online food delivery"[Title/Abstract] | 26 |
| 2 | "Food delivery apps"[Title/Abstract] | 7 |
| 3 | "Food online"[Title/Abstract] | 14 |
| 4 | "Food delivery service"[Title/Abstract] | 13 |
| 5 | "Internet food"[Title/Abstract] | 3 |
| 6 | "Digital food environment"[Title/Abstract] | 6 |
| 7 | "On-demand food"[Title/Abstract] | 0 |
| Alcohol |  |  |
| 8 | "Alcohol online"[Title/Abstract] | 8 |
| 9 | "Internet alcohol"[Title/Abstract] | 8 |
| 10 | "Alcohol delivery"[Title/Abstract] | 18 |
| 11 | "Alcohol website"[Title/Abstract] | 4 |
| 12 | "On-demand alcohol"[Title/Abstract] | 0 |
| 13 | "Alcohol home delivery"[Title/Abstract] | 4 |
|  | **Total records identified** | **111** |

| **Scopus (Elsevier)** | |  |
| --- | --- | --- |
| # | Search Syntax | 7/03/2022 |
| Food |  |  |
| 1 | TITLE-ABS ("Online food delivery") | 170 |
| 2 | TITLE-ABS ("Food delivery apps") | 57 |
| 3 | TITLE-ABS ("Food online") | 101 |
| 4 | TITLE-ABS ("Food delivery service") | 199 |
| 5 | TITLE-ABS ("Internet food") | 15 |
| 6 | TITLE-ABS ("Digital food environment") | 6 |
| 7 | TITLE-ABS ("On-demand food") | 39 |
| Alcohol |  |  |
| 8 | TITLE-ABS ("Alcohol online") | 13 |
| 9 | TITLE-ABS ("Internet alcohol") | 11 |
| 10 | TITLE-ABS ("Alcohol delivery") | 16 |
| 11 | TITLE-ABS ("Alcohol website") | 5 |
| 12 | TITLE-ABS ("On-demand alcohol") | 2 |
| 13 | TITLE-ABS ("Alcohol home delivery") | 5 |
|  | **Total records identified** | **639** |

| **Embase (OVID interface, 1947+)** | |  |
| --- | --- | --- |
| # | Search Syntax | 7/03/2022 |
| Food |  |  |
| 1 | "Online food delivery".ti,ab. | 18 |
| 2 | "Food delivery apps".ti,ab. | 5 |
| 3 | "Food online".ti,ab. | 14 |
| 4 | "Food delivery service".ti,ab. | 16 |
| 5 | "Internet food".ti,ab. | 6 |
| 6 | "Digital food environment".ti,ab. | 5 |
| 7 | "On-demand food".ti,ab. | 0 |
| Alcohol |  |  |
| 8 | "Alcohol online".ti,ab. | 7 |
| 9 | "Internet alcohol".ti,ab. | 12 |
| 10 | "Alcohol delivery".ti,ab. | 40 |
| 11 | "Alcohol website".ti,ab. | 4 |
| 12 | "On-demand alcohol".ti,ab. | 2 |
| 13 | "Alcohol home delivery".ti,ab. | 4 |
|  | **Total records identified** | **133** |

**Supplementary Table 3 – Project-specific charting table used to guide data extraction**

| **Item** | **Description** |
| --- | --- |
| Author(s) |  |
| Title |  |
| Year of publication |  |
| Type of publication | Journal article, Report, Conference proceedings, Pre-print |
| Journal name | If relevant |
| Funding | How was study funded? Research grant? Industry grant? |
| Country/geographical area | For example: By country, By geographical area, By income category (high, middle, low-income countries) |
| Aims/objectives | The objective of the publication as stated |
| Commodity type | Food, Alcohol, Both |
| Role within on-demand services | Consumers, Delivery drivers or other employee, Commodity providing services, Other / multiple |
| Study population | For example: Men, Women, Children, Adolescents, Elderly, By level of income, Deprivation (area and SES), Ethnicity, Websites, Businesses / services |
| Sample size | N’s of study population |
| Methods | For example: Survey, Website content analysis, Interviews, Geospatial, App-usage data, Others as stated in the study |
| Health/public health outcomes | Describe the reported health/public health outcomes for studies, including how these were measured |
| Other outcomes | Describe other reported outcomes |
| Regulatory or policy outcomes (from a health perspective) | Describe the reported regulatory or policy outcomes from the studies |
| Findings | Describe findings of study |
| Limitations | Describing the limitations of the study (as outlined in the study) |

**Supplementary Table 4 – Summary of extracted characteristics and findings from across the 40 included studies**

| **Reference** | **Geographical area** | **Role within on-demand services** | **Study population** | **Aims / objectives** | **Methods** | **Outcome measure/s** | **Main findings** | **Limitations** |
| --- | --- | --- | --- | --- | --- | --- | --- | --- |
| **Commodity type: Food** | |  |  |  |  |  |  |  |
| [Allen et al. (2021)](#_ENREF_1) | London, UK | Commodity providing services | Meal delivery data from a food delivery provider (n= 40,941 deliveries) | Understand operational performance of meal deliveries by drivers using bicycles, mopeds, and cars and the transport and environmental impacts | Spreadsheet-based data analysis to quantify greenhouse gas (GHG) emissions of vehicle activities and transport intensity | **Health**: Transport intensity (kerb occupancy, distance travelled) and GHG emissions | - Meal deliveries were relatively transport inefficient compared to other forms of urban road freight (due to the delivery being dedicated to a small quantity of goods - Meals delivered by car and petrol moped have far greater GHG emissions and transport intensity compared to bicycles | - Based on a single on-demand platform in a single city, over one time period |
| [Arunan and Crawford (2021)](#_ENREF_2) | Australia | Commodity providing services | Adult users of online food delivery services aged 18-45 (n=73); Restaurant managers (n=19); Food packaging (n=5 cuisines; Pizza, Indian, Thai, Chinese, Burger) | Quantify packaging related GHG emissions associated with online food delivery orders through Uber Eats | Consumer survey. Interviews with restaurant managers to determine commonly ordered items and packaging. Life cycle assessment to determine GHG emissions. Food packaging assessed using the Packaging Impact Quick Evaluation Tool (PIQET) | **Health**: Online food delivery use and packaging disposal practices; GHG emissions of packaging production and disposal  **Regulatory**: Annual GHG emissions (projected to 2024) | - With uncontaminated packaging, 84% disposed of packaging for recycling and 16% into landfill - With contaminated utensils, washing to reuse and disposing to landfill were most common disposal methods; with contaminated packaging, washing to recycle and washing to reuse were more common - Production of raw packaging materials contributes at least 50% to the GHG emissions for a single order - The annual GHG emissions associated with online food delivery packaging is predicted to grow by 132% by 2024 | - Did not consider emissions associated with food delivery - Potential gaps from incomplete inventories of packaging materials - Limitations in the embedded inventory data in PIQET software (used to model emissions) |
| [Babar et al. (2021)](#_ENREF_3) | 85 counties in the USA | Consumers | Participants in the American Time Use Survey across 85 counties | Determine impacts of Grubhub (online food delivery platform) introduction on food consumption habits | Survey data aggregated into county data (domestic cooking behaviours, in-restaurant dining, BMI data), before and after introduction of Grubhub (2005-2019). Sampling from Grubhub website | **Health:** Time spent on meal preparation,  Time spent on dining in restaurants, BMI | - In counties with Grubhub entry, daily meal preparation reduced by an average of 4.5 minutes compared to the pre-treatment average - Time spent dining in restaurants was not significantly different after Grubhub entry - Entry of the delivery service is associated with a 0.5% increase in average population BMI | - Geographic pooling, rather than individual behaviour or outcomes - Assumptions: uniform availability across county; at-home meal prep being healthier than food available on-demand |
| [Björkbom and Nguyen (2021)](#_ENREF_5) | Stockholm, Sweden | Commodity providing services | Consumers of food delivery services (n=35) | Examine emissions impacts of last mile online-food deliveries in the context of the goal for Stockholm to become fossil-free by 2040 | Two quantitative surveys of consumers focused on the services that they used; one qualitative interview with Head of Sustainable Transformation at PostNord | **Health**: Environmental impacts (delivery method, distance travelled, vehicle type)  **Other**: Difference in mode shown on app and in reality | - Wide use of delivery modes that use fossil fuels - Discrepancies noted between what the companies listed as primary transport mode (e.g., bicycle) and the actual mode (e.g., moped or car) - 54% of travel distances were 2-4 km from restaurant to delivery point | - Small sample size |
| [Brar and Minaker (2021)](#_ENREF_6) | Ontario, Canada | Commodity providing services | Retailers appearing on DoorDash app (n=480; incorporating 20 retailers from each of 24 randomly selected urban post codes) and menu items of 12 retailers from four popular online food delivery apps (n=759) | Examine the extent to which online food delivery services geographically expand retail food environments and evaluate the healthfulness of foods available | Distance to first/last ten retailers in each postal code examined in relation to city population, population density, and first/last appearance in app.  Menus coded using the Food and Nutrient Database for Dietary Studies-2015 and Food Patterns Equivalents Database-2015 | **Health**: Online food delivery and geographic food access; healthfulness of online food delivery menus determined through Healthy Eating Index-2015 (HEI-2015) scores | - Online food delivery services substantially increase geographic access to foods prepared away from home (by up to 9.4 km and 472 retailers) - Distance to the retailer may factor into how online food delivery apps display retailers - HEI-2015 scores for retailers’ full menus were typically low, indicating food offerings do not meet healthy eating recommendations | - Randomly selected urban postal codes do not reflect work locations - Summing dietary constituents across menus does not capture variability across individual items or combinations - Does not assess children's menus |
| [Eu and Sameeha (2021)](#_ENREF_13) | Malaysia | Consumers | Students 19-29 years from 20 public universities (n=290) | Determine online food delivery app consumers' perceptions of availability of healthy food in apps and the association with food choices | Online questionnaire | **Health**: Perceptions of healthy food availability; app-based food choices  **Other**: Factors affecting consumer food choice | - Most consumers had a negative perception of healthy food availability (variety, price, quality of healthy food) in online food delivery apps - No significant associations between perceptions of healthy food availability and consumer food choices (healthy or unhealthy) - Price and convenience of food was the greatest influencing factor on food choices | - Retrospective cross-sectional study based on experiences before COVID-19 restrictions - Higher number of Chinese participants and those from one university - No appropriate reference to classify foods as healthy or unhealthy |
| [Gregory (2021)](#_ENREF_18) | Edinburgh, Scotland | Employees | On-demand food couriers aged 18-45 years (n=25 current or former) | Understand how riders take up and experience on-demand, app-based food courier work in Edinburgh, and how they construct and represent work-related risks | In-depth interviews | **Health**: Risks (bodily risk and physical harm, financial risks, epistemic risks) | - Platformed labour creates a range of risks, including physical risk and bodily harm (road accidents, harassment), financial risks (earnings and unpredictable wages) and epistemic risks (algorithmically managed work creates uncertainty and obscures allocation processes and associated risks) - To negotiate risks, couriers privatise, normalise, and minimise risks and forging new communities of support | - Conducted before the COVID-19 pandemic which has exacerbated physical, health, safety, and financial risks faced |
| [Hasegawa et al. (2022)](#_ENREF_20) | Japan | Employees | Uber Eats delivery persons (n=188) | Examine food delivery service gig workers who worked during the COVID-19 recession and to understand the labour supply of Uber Eats delivery persons | Survey of drivers | **Health**: Worker income | - Those who lost their job during the pandemic and were lower income earners entered the gig economy during the pandemic - At least one-third of drivers worked at an hourly wage below minimum wage - Discussion on protecting gig economy workers health and safety | - Small sample size - Focused on Uber Eats delivery persons - Pandemic may have impacted findings |
| [Hodges Jr (2020)](#_ENREF_23) | Texas, USA | Employees | Delivery couriers aged 18 years or over (n= 883 survey responses, n=10 interviews) | Understand food safety behaviours and knowledge of online food delivery couriers | Survey and structured interviews | **Health**: Self-reported food safety knowledge and behaviours, causes of stress | - Unsafe behaviours included insufficient handwashing and taking undelivered food home for personal consumption - Average score on the food safety knowledge assessment was 69.6% - Interviewees reported a need for more food safety training, significant stress caused by pressure to maintain rating scores, and no requirement or checking that drivers wash their hands regularly | - Self-reported data and self-selected sample |
| [Horta et al. (2020)](#_ENREF_26) | Minas Gerais, Brazil | Commodity providing services | Commercial food establishments (n=362) offering services through two apps | Examine food availability and use of marketing strategies by two food delivery apps in a Brazilian metropolis | Menus and prices extracted from apps. Ten best rated restaurants sampled from each app, across 18 administrative regions | **Health**: Health profile of food groups  **Other**: Discounts offered | - Ultra-processed meals represented almost 70% of food offered - Marketing strategies and discounts were directed predominantly towards ultra-processed and discretionary items, not fruits and vegetables | - Sample was of best-rated restaurants, not all - Some foods could not be easily classified (e.g., Japanese food) |
| [Horta et al. (2021)](#_ENREF_24) | All 27 capital cities of Brazil | Commodity providing services | Random 25% sample of advertisements on a single app (n=1754) | Describe advertisements published in an online food delivery platform in Brazilian capitals, during the 13^th^ and 14^th^ weeks of the COVID-19 pandemic | Ads were classified by the presence of healthy and unhealthy eating markers and the use of marketing strategies | **Health**: Health profile of advertised food  **Other**: Marketing strategies integrated within the ad | - Healthy food was advertised more during the week and at lunchtime, with unhealthy food advertised more during weekend and dinner time - Free delivery was marketed more with discretionary food items, and marketing strategies were directed more to ads containing unhealthy foods | - Conducted in early stages of the pandemic |
| [Horta et al. (2022)](#_ENREF_25) | Minas Gerais, Brazil | Commodity providing services | Sample of app-based food promotions (n=1593; 25% of 6372 advertised food items identified) | Analyse food advertised on an online food delivery platform during 16 weeks of the COVID-19 pandemic in Brazil | Data collected from online food delivery app home page. Food items classified into food groups and as healthy/unhealthy. Marketing items and strategies quantified and characterised. | **Health**: Healthiness of food being offered according to NOVA food classifications and Brazilian Ministry of Health Dietary Guidelines  **Other**: Marketing strategies | - During the 16 week period, app-based food promotions primarily featured unhealthy foods and beverages - Sandwiches, pizza, and ultra-processed beverages were promoted more frequently than water, natural juices, smoothies, vegetables, and fruit - Marketing strategies included photos, discounts, claims about tastiness, pleasure, and value for the money | - Only studied the foods advertised on the home page of one platform - Could not evaluate changes in digital food environment after restaurants/bars reopened |
| [Huang (2021)](#_ENREF_27) | China | Employees | Delivery drivers from top Chinese food-delivery platforms aged 18 years or over (n=52). Most drivers relied on the food-delivery work as their sole source of income. | Examine the impact of COVID-19 on platform-based food-delivery drivers in China, particularly focusing on labour conditions | Online semi-structured interviews. Data supplemented by news, opinions, statistics, business report from food-delivery platforms | **Health**: Labour conditions (including income, perceptions of work during pandemic) | - Precarity of drivers’ work and life is dramatically amplified by the pandemic and reorganisation of algorithmic labour process - Work security: Drivers struggle with increased physical risks (traffic accidents, COVID-19 risk/infection, race algorithms to deliver food on time by exposing themselves to more environmental) - Income stability precarity: Drivers classified as independent contractors (denied access to pandemic-related benefits and support); COVID-19 restrictions mean drivers cannot leave their homes to work in the city; drivers responsible for purchasing own PPE - Racialised precarity: Drivers labelled as 'virus carriers' | - Not stated |
| [Jia et al. (2018)](#_ENREF_31) | China | Commodity providing services | Modelling of all online food deliveries | Investigate waste, energy consumption, and emission issues related to the increase of online food deliveries in China | Analysis of the energy consumption and emissions from online food packaging waste generation and delivery using modelling | **Health:** Waste generation, energy consumption, and GHG emissions | - Waste from food deliveries is remarkable and increasing | - Only considered waste generated during delivery, not the whole production chain |
| [Jia et al. (2021)](#_ENREF_30) | Australia, UK, USA | Commodity providing services | Publicly available Instagram accounts (n= 581 posts) for three top online delivery services in Australia (Uber Eats, Menulog, Deliveroo), UK (Just Eat, Uber Eats, Deliveroo), USA (DoorDash, Uber Eats, GrubHub) | Explore promotion of discretionary foods/beverages and marketing strategies used in Instagram accounts of three top online delivery services from Australia, UK, USA, before (2019) and during (2020) the COVID-19 pandemic | Content analysis of Instagram accounts. Food/beverage items from posts were classified as 'discretionary' or from the 5 Food Groups according to the Australian Dietary Guidelines | **Health**: Nutritional quality of featured foods/beverages  **Other**: Marketing strategies | - Instagram posts increased during the pandemic, with a larger proportion portraying discretionary food/beverage items (69%) compared to pre-pandemic posts - Three accounts featured discretionary food/beverage items in over 90% of food-related posts - COVID-19 marketing strategies included: combatting the pandemic; selling social distancing; appropriating frontline workers; accelerating digitalisation - Many food/beverage items featured in COVID-19-related posts were discretionary items (over 97% of COVID-19 posts in Australia and UK featured discretionary foods) | - Only considered marketing on one form of social media - Only considered posts made directly to Instagram page (not user-generated content, paid advertising, influencers, or targeted advertising) |
| [Keeble et al. (2020)](#_ENREF_33) | Australia, Canada, Mexico, UK, USA | Consumers | Adult users of online food delivery services aged 18 years or over (weighted by age and sex; n=2929) | Describe prevalence and frequency of online food delivery service use, associations between use and sociodemographic characteristics, and how customers used other modes to purchase food away from home, in and across upper-middle or high-income countries | Secondary analysis of survey data | **Health**: Self-reported BMI  **Other**: Demographics of use, between country variance | - No difference in prevalence of use between BMI categories - Users of on-demand services more likely to be male, ethnic minority, younger, higher education, and living with children under 18 years - The findings correlate similarly between countries | - Self-reported data - Unable to determine type of food ordered - Education used as a marker of SES |
| [Keeble et al. (2021a)](#_ENREF_32) | England | Commodity providing services | Postcode districts in England (n = 2118; 29,232 food outlets registered to accept online food delivery orders) | Describe access to food outlets and cuisine types through Just Eat online food delivery service across England; compare online and physical food outlet access within neighbourhoods; and examine associated with deprivation | Web-browser extension used to collect data on food outlets accessible through Just Eat. Neighbourhood comparisons made over 1600 m Euclidean buffers of postcode district geographic centroids | **Health:** Number of food outlets and cuisine types accessible online  **Other**: Neighbourhood deprivation | - A median of 64 food outlets and 39 unique cuisine types were accessible online per postcode district - A median of 63% of food outlets accessible online were accessible within the neighbourhood - Online food outlet access in England is socioeconomically patterned - Online food outlet access was highest in the most deprived postcode districts - The percentage of food outlets accepting orders online increased with deprivation - The number of food outlets accessible online (as a percentage of those accessible within the neighbourhood) was highest in the least deprived postcode districts | - Food outlets registered with Just Eat but not identified during data collection were excluded - Postcode districts limited to boundary data from 2012 - 1600 m straight-line buffer used to define neighbourhood |
| [Keeble et al. (2021b)](#_ENREF_34) | UK | Consumers | Adults aged 18 years or over (n=3067) | Investigate association between aspects of online food outlet access and use, differences according to customer sociodemographic characteristics, and associations between number of outlets accessible online and bodyweight | Web-browser extension used to collect data on food outlets accessible through Just Eat (n=33,204). Food outlet data linked with food purchasing, bodyweight, and sociodemographic data. Neighbourhood comparisons made over 1600 m Euclidean buffers of postcode district geographic centroids | **Health**: Online food outlet access and online food delivery service use  **Other**: Self-reported BMI | - UK adults had online access to a median of 85 food outlets and 85 unique cuisines - Adults with the greatest number of accessible food outlets had 71% greater odds of reporting online food delivery service use, compared to those with the least access - No association between number of unique cuisines accessible online and online food delivery service use, or between the number of food outlets accessible online and bodyweight | - Data pre-dates changes in food purchasing practices due to COVID-19 - Food outlets registered with the online food delivery service but not identified during data collection were excluded - Cross-sectional analysis and self-report survey data - 1600 m straight-line buffer used to define neighbourhood |
| [Liu et al. (2020)](#_ENREF_38) | Jing-Jin-Ji region, China | Commodity providing services | Packaging/utensils from over 200 food delivery service providers | Evaluate the impact of urban food delivery service packaging on the environment and clarify the overall environmental burdens of the urban food delivery service sector in the Jing-Jin-Ji region | Life cycle assessment across five categories of packaging, using a big data approach | **Health**: Environmental impact categories including greenhouse effect, carcinogen toxicity, ecotoxicity | - Food packaging accounts for 15.7% of the total municipal solid waste in the area - Greenhouse effect is the most substantial environmental impact - Paper boxes contribute the most environmental pollution | - Health impacts were modelled rather than being directly measured |
| [Maimaiti et al. (2020)](#_ENREF_39) | Hangzhou, China | Commodity providing services | Food outlets (n=9274) | Categorise food outlets in the Xi Hu district and identify characteristics and trends of the Chinese food environment | Direct observation and survey of food outlets using standardised forms | **Health**: Healthiness of food categorised by BMI-healthy, BMI-intermediate, and BMI-unhealthy | - Fast food restaurants had the highest proportion of also providing on-demand delivery (66%), compared to other outlet types - In total, 42% of food outlets offered on-demand delivery | - May not be representative of all China |
| [Martha et al. (2021)](#_ENREF_40) | Depok City, Indonesia | Consumer | Young adults 20-39 years (n=686) | Investigate factors influencing frequency of online food ordering behaviour and consumption of high-risk foods | Cross-sectional survey | **Health:** Consumption of high-risk foods (high-sugar and high-fat type of food items) | - Consumers influenced by socio-environmental factors and high preference for using online food delivery services exhibited significantly increased high-risk food consumption through online food delivery services than those who did not | - Self-reported consumption via a survey - Cross-sectional survey |
| [Partridge et al. (2020)](#_ENREF_47) | Australia, New Zealand | Commodity providing services | Unique food outlets offered through Uber Eats (n=1074) and popular menu items (n=5769) | Evaluate healthiness and geographical reach of popular food outlets and nutritional quality of menu items on an online food delivery platform, in areas with high concentrations of young consumers. Secondary aim to examine differences in outlet characteristics and SES | Data extracted from Uber Eats across various areas. Nutritional quality of food outlets assessed as healthy/unhealthy using the Food Environment Score (FES). Most popular items classiﬁed as discretionary or core according to Australian Dietary Guidelines | **Health**: Healthiness of outlets by FES score, and food items categorised as discretionary or core  **Other**: Deprivation quintiles | - Almost 3/4 of food outlets scored as unhealthy in both cities - Almost 90% of popular menu items were categorised as discretionary food - There were more outlets in Sydney in least disadvantaged suburbs, compared to an even spread across Auckland - There were SES differences in healthiness in Auckland, with more healthy outlets in less disadvantaged areas - There were no differences by proportion of discretionary food items | - Potential overestimation of core foods - Only one platform analysed |
| [Poelman et al. (2020)](#_ENREF_51) | USA (Chicago), Netherlands (Amsterdam), Australia (Melbourne) | Commodity providing services | Meal delivery service websites across three cities (n=4323 restaurants) | Explore online meal delivery exposure, advertised types of food, and pricing in three international cities within high-income countries and  compare these dimensions by neighbourhood socioeconomic disadvantage | Multiple address extraction and sampling (stratified by socioeconomic disadvantage). Restaurant keyword analysis | **Health**: Types of food available by categories  **Other**: Price of food, addresses stratified by SES | - Advertised foods were typically unhealthy - Demographically, there was less advertisement of healthy meals in Chicago and Melbourne vs. Amsterdam - In all three areas, the type of food offered varied with disadvantage - Price variance by neighbourhood only occurred in Chicago and Amsterdam | - Looked at keywords not actual menus - Did not examine nutritional characteristics |
| [Poelman et al. (2021)](#_ENREF_50) | Netherlands | Consumer | Adults aged 18 years or over (n=1030) | Assess changes in eating behaviour and food purchases five weeks into the COVID-19 lockdown | Secondary analysis of survey data | **Health**: Use of on-demand meal delivery services due to lockdown  **Other**: Types of food purchased | - Use of services and dietary habits did not substantially change due to lockdown. Of those who used meal delivery services pre-lockdown, 49.7% used meal-delivery services as usual during lockdown, 29.5% used them more frequently, and 20.7% used them less frequently - Those self-reporting as obese were more likely than those of a healthy weight to order pizza or deep-fried food more often than usual | - Self-reported survey |
| [Sarkies et al. (2021)](#_ENREF_53) | Sydney, Australia | Employees | Adults aged 18 years or over presenting to an acute public hospital ED after a cycling-related injury (n=368) | Determine whether cyclists could be categorised as commercial or non-commercial from routine medical records and which key demographic, incident, and injury characteristics could be attributed to each category | Retrospective review of medical records | **Health**: Injuries sustained by delivery cyclists | - 11.7% of injuries could be identified as commercial cyclists - Commercial cyclists had a lower mean age and fewer spoke English as their primary language, compared to other cyclists - Commercial cyclists were more likely to be struck by a motor vehicle than other cyclists | - A substantial proportion of presentations were unable to be categorised as commercial or non-commercial - Medical records limited to a single site over a 12-month period |
| [Tan et al. (2021)](#_ENREF_57) | China | Employees (road accidents cannot necessarily be attributed to delivery drivers) | Data from 18 Chinese cities across 2007-2019 | Examine benefits (unemployment rates) and costs (road accidents statistics, death counts) of online food delivery resulting from the entry of two platforms (Meituan, Eleme) into China | Staggered difference-in-difference methodology to determine causal effects | **Health**: Road accidents, death counts  **Other**: Unemployment rates, estimated economic costs of road accidents | - The arrival of online food delivery services caused an average increase of 611 road accidents and 51 deaths per city per year, but had no significant effect on unemployment rates - Estimated economic costs of road accidents following the entry of online food delivery services is approximately 3.65 million CNY per city each year | - Short term utility of results given the recent emergence of online food delivery in China - Small city sample size |
| [Thompson et al. (2022)](#_ENREF_58) | California, USA | Commodity providing services | Low income neighbourhoods in California (226 census tracts); 631 observations from 254 on-demand delivery platforms from 13 chains | Examine consistency with Healthy-By-Default Beverage Law (SB1192; mandating only water or unflavoured dairy/non-dairy milk be default drinks with children’s meals) for meals sold through online platforms from restaurants in low-income neighbourhoods | Cross-sectional sampling of on-demand delivery restaurants selling children's meals | **Health**: Healthiness of children's beverages, beverage availability, upcharges (additional cost)  **Regulatory**: Compliance with California’s Healthy-By-Default Beverage law (SB1192) | - Seventy percent of observations offered water; 63% offered unflavoured milk - Among all beverages, water was most likely to have an upcharge; no upcharges for soda were observed - Most on-demand delivery restaurants are not offering children’s meal beverages consistent with the state law | - No data available for a pre-post comparison following the law change |
| [C. Wang et al. (2021)](#_ENREF_62) | Sydney, Australia | Commodity providing services | Popular food outlets extracted from Uber Eats (n=196 independent takeaways; n=13841 menu items) | Evaluate nutritional quality of complete menus and marketing attributes of offerings from independent takeaway outlets available on the market-leading online food delivery platform (Uber Eats) | Complete menus and marketing attributes collected using web scraping. Menu items classified into 38 food and beverage categories. Most popular food outlets extracted from the “popular near you” section | **Health**: Nutritional quality of complete menus based on Australian Dietary Guidelines’ Five Food Group (FFG) and discretionary classifications  **Other**: Marketing attributes for complete menus | - Complete menus consisted of predominantly unhealthy choices: 80.5% were discretionary and 42.3% were discretionary cereal-based mixed meals - Discretionary mixed meals (cereal-based and vegetable-based) were more expensive than their FFG counterparts - Discretionary menu items were more likely to be categorised as ‘most popular’, accompanied by an image, and offered as a value bundle compared to FFG | - Findings cannot be generalised to all independent takeaways - Limited to marketing attributes available through the web browser and not the mobile application interfaces |
| [X. Wang et al. (2021)](#_ENREF_63) | China | Commodity providing services, Consumers | Adults, children, pregnant women (n=952 questionnaires from 32 provinces); food packaging (n=18) | Investigate phthalic acid esters (PAEs) commonly found in plastic takeaway containers in the Chinese market and the health risks for frequent consumers of food from disposable plastic containers | Questionnaire used to determine takeaway habits and exposure parameters for population health risk assessment. Based on the migration of PAEs to the food from the plastic packaging, chronic daily intake, hazard quotient, and carcinogenic risk calculated for different regions and populations | **Health**: Health risks for people who frequently consumed food from disposable plastic containers | - Health risks from PAEs exposure were within acceptable limits and no significant health risks were identified - High food temperatures, high-fat food characteristics, and higher frequency of consumption will exacerbate the health risks | - Small sample of pregnant women - Only considered four PAEs - Mobility of PAEs calculated at 60°C, but food containers often come into contact with higher temperatures |
| [Zhang et al. (2020)](#_ENREF_70) | China | Consumers | Adult residents 18-80 years (n=1994) | Observe changes in dietary behaviour of Chinese residents during the COVID-19 pandemic and to explore the potential health impacts | Online survey conducted during the post-lockdown period | **Health**: Household Dietary Diversity Scores (HDDS; reflects household food accessibility) | - Those who increased consumption of online food delivery during the pandemic had higher HDDS, even after adjustment for family income and location | - Nutrient intake not assessed - Online survey may have missed some demographics |
| [Zhang et al. (2022)](#_ENREF_69) | Wuhan, China | Commodity providing services, Consumers | Food delivery waste samples (n=810); Consumers (white-collar workers, college students, urban residents; n=889) | Characterise total and per capita food delivery waste generated by different consumer groups, identify driving factors of food delivery waste generation, and analyse related carbon emissions and explore mitigation strategies | Food delivery waste (avoidable (edible food), unavoidable (inedible food), packaging waste) sampled from four colleges, three office buildings, and three residential areas. Life cycle assessment across transportation (to consumers/disposal sites) and disposal. Three scenarios developed to explore potential reductions in carbon emissions. | **Health**: Quantify food delivery waste and carbon emissions  **Other**: Consumer behaviours, awareness, and attitude toward food delivery waste | - Avoidable food waste made up the largest share of total waste (55%, compared to 13% unavoidable matters) - Packaging waste accounted for 32% of the total food delivery waste and had an overwhelming role in total carbon emissions due to the amount produced - Males under 35 years generated the most waste - White-collar workers and students with higher education generated more waste compared to urban residents - Food delivery waste disposal presented the largest share of carbon emissions (75%), followed by food and waste transportation - Emissions can be reduced by 55% if the avoidable food waste can be prevented | - Sampling sites and individual samples were selected from three consumer groups - Samples were primarily collected from garbage bins and measured by direct weighing, not considering possible mixture with other waste |
| [Zhao et al. (2020)](#_ENREF_71) | China | Consumers | Adult residents 18-80 years (n=2021) | Assess dietary diversity among Chinese residents during the time of isolation and lockdown due the COVID-19 pandemic | Online survey conducted during COVID-19 restrictions | **Health**: Household Dietary Diversity Scores (HDDS; reflects household food accessibility) | - There was no difference in dietary diversity between those who typically used online food delivery compared those who did not | - Convenience sample - Did not seek to quantify actual food intake - HDDS does not measure access to food |
| [Zhou et al. (2020)](#_ENREF_72) | China | Commodity providing services | City-level takeaway data collected from Meituan over a six month period in 2018 (National packaging consumption of 353 cities; 2.8 billion takeaway orders) | Use a top-down approach with city-level takeaway order data to explore the packaging waste and life-cycle environmental impacts of the takeaway industry in China | Life-cycle environmental impacts of takeaway industry estimated under three scenarios (current packaging and waste disposal patterns; paper substitution; tableware sharing) | **Health**: Waste generation; environmental impacts (water consumption, emissions) of takeaway packaging waste  **Other**: Mitigation strategies | - Single-use food containers, plastic bags, and tissues have higher environmental impacts than other takeaway packaging - Raw material production is the major source of CO_2_ emissions, followed by incineration - Tableware sharing could reduce waste generation by up to 92%, and environmental emissions and water consumption by more than two-thirds, compared to current patterns | - Type, material, size of tableware and packaging were simplified - Only used data from Meituan platform - Only focused on environmental impacts of takeaway packaging, and excluded food waste |
| **Commodity type: Alcohol** | | |  |  |  |  |  |  |
| [Colbert et al. (2020)](#_ENREF_9) | Australia | Commodity providing services | Most popular online alcohol retailers providing alcohol delivery (n=65) | Provide information about advertised sales/delivery practices of online alcohol retailers and potential implications for alcohol-related harm | Website content analysis of sales and delivery practices | **Health**: Costs of provided alcohol  **Regulatory**: Policy statements (age warnings, liquor license details, refusing delivery to intoxicated persons)  **Other**: Website traffic, product range, promotional activity, age verification, payment and delivery | - Most retailers sold low cost alcohol (some under $5AUD per bottle of wine) and multiple alcohol types - Low cost warehouse style liquor outlets were the most popular - Most retailers offered opportunities to receive promotional material and bulk buy discounts - 13.8% of retailers allowed purchases through ‘buy now, pay later’ schemes - Most had an age warning and half required users to enter a date of birth - 75.4% of retailers would leave alcohol unattended on delivery - Most retailers displayed liquor licence numbers, all mentioned it is illegal to sell alcohol to those under 18, and 20% mentioned refusal of delivery to intoxicated persons | - Only captures information at one point in time - Popularity of website based on estimated visitors in one month - Unable to assess how practices are implemented |
| [Colbert et al. (2021)](#_ENREF_10) | USA, Canada, UK, Ireland, Australia, New Zealand | Commodity providing service | Six English-speaking OECD countries covering 77 jurisdictions | Summarise international policies governing online alcohol sale/delivery and any COVID-19 related changes | Policy review  according to ten elements relevant for public health regulation. Policies identified through databases and official legislative and regulatory websites. | **Regulatory**: Policies to prevent youth access to alcohol home deliveries (age verification at purchase/delivery; alcohol labelling; delivery driver training; age warnings, liquor license details on websites). Policies around delivery (types/quantities; type of premise permitted to deliver; trading hours; third party delivery services). | - Most jurisdictions (94%) permitted online alcohol sales and delivery - Few jurisdictions (9%) required age verification at purchase, while 92% required it at delivery - Only 21% of jurisdictions permitting alcohol delivery required drivers to be trained in responsible alcohol service - Some jurisdictions (USA, Canada) have quantity limits on alcohol delivery, either per transaction or month/year - Seven jurisdictions permit alcohol deliveries outside maximum trading hours for licensed premises - Few jurisdictions required age warnings or liquor license details to be shown on websites (contrasting to physical store requirements) - Since the pandemic began, 69% of jurisdictions have relaxed regulations for alcohol home delivery; 13 jurisdictions have made permanent changes - Policy recommendations provided. | - License categories broadly grouped into on- and off-premises - Only considered state-wide policies (local government policies may be missed) - Did not examine implementation or enforcement of policies |
| [Huckle et al. (2020)](#_ENREF_28) | New Zealand | Consumers | Adults aged 18 years or over (n=2173) | Assess purchasing and drinking behaviour in the context of the COVID-19 pandemic restrictions | Online survey (delivered via Facebook) during pandemic restrictions April/May 2020 | **Health**: Pattern of drinking (i.e., heavy drinking) and quantity drunk per occasion  **Regulatory**: Reliability of ID verification | - Online alcohol delivery during pandemic restrictions was associated with heavier drinking in the past week - Consumers using online delivery services had 75% higher odds of heavy drinking, and 24% higher typical occasion quantity - 57.5% of consumers aged <25 years were not asked for age verification on delivery - 15.5% of consumers reported running out of alcohol while drinking and ordering more online, with 7% receiving delivery in less than 3 hours | - Results weighted to females - Convenience sample |
| [Mojica‐Perez et al. (2019)](#_ENREF_43) | Australia | Consumers | Adults aged 18 years of over who have used online alcohol delivery services within the last month (n=528) | Examine characteristics of alcohol delivery service users, purchasing and drinking practices, and reasons for using the services | Online survey | **Health**: Alcohol use in last 12 months  **Other**: Usage patterns of alcohol delivery services, reasons for using delivery services | - The majority (59.5%) of respondents bought alcohol online from a bottle shop and 15.9% reported using specialised fast delivery companies (e.g., Uber Eats) - More than a third used an alcohol delivery service at least monthly - Respondents using fast delivery services reported higher median drink prices and lower median expenditure. 57% reported their choice of restaurant was influenced by availability of alcohol - Reasons for using fast delivery services included convenience, not wanting to leave the home, being over the blood limit for driving - Fast delivery services were most likely to be used between 6pm and 12am and by 18-29-year olds - 36.2% of respondents aged <25 years did not have their ID checked - 69.2% of respondents who ordered on-demand drank 5+ standard drinks on that occasion (28.6% had 11+ drinks) - Over a quarter of respondents who received an on-demand order stated they would have had to stop drinking alcohol if the delivery service was not available - For respondents whose last order was on-demand, they generally drank more heavily than those whose last order took longer than two hours | - Convenience sample |
| [Noyes et al. (2021)](#_ENREF_46) | New South Wales, Australia | Commodity providing services | Online liquor retailers in New South Wales (NSW) with publicly available websites (n=213 retailers) | Determine the extent to which online liquor retailers have adopted safeguards to prevent young people (<18 years) and intoxicated people purchasing alcohol | Audit of regulatory controls for online liquor retailers with publicly available websites. NSW Liquor Act 2007 and the NSW Liquor Regulation 2018 were reviewed to assess the regulatory framework protecting against supply of alcohol to underage or intoxicated people | **Regulatory**: Discrepancies between safeguards that apply to online and physical liquor retailers. The extent that online retailers have adopted existing safeguards (mandatory signage, age verification, purchasing and supply) | - Three gaps in the existing legal safeguards for online liquor retailers identified: inconsistent application of the Liquor Act 2007 (particularly, around verifying age at point of supply); inability of the regulator to audit compliance; and absence of consistent mandatory signage - Inconsistencies between regulation of physical and online retailers - 22% of retailers specified delivery could only be completed if someone >18 years accepted the goods - Only 40% of retailers required date of birth to be supplied at purchase - Only 5% of retailers included policy on their website about delivery to intoxicated persons - Policy recommendations provided. | - 1/3 of active online liquor licenses were unable to be located at the time of the audit - Desktop audit of publicly available information and policies; no physical audit of company behaviours |
| [Van Hoof et al. (2015)](#_ENREF_60) | Netherlands | Commodity providing services | On-premise, off-premise, and online alcohol vendors (n=2,737 underage alcohol purchase attempts) | Measuring vendors compliance and change in compliance with legal age limits on alcohol sales | Representative mystery shopping studies in 2011 and 2013 | **Regulatory**: Underage access to alcohol | - 53.5% of adolescents were able to purchase alcohol across the various vendors - For online sales, age verification and compliance with age limits for alcohol sales were not met and did not improve between 2011 and 2013 | - Not stated |
| **Commodity type: Food and Alcohol** | | |  |  |  |  |  |  |
| [Miles et al. (2022)](#_ENREF_42) | New Zealand | Commodity providing services | On-demand services in New Zealand (n=130) | Quantify how many services provide on-demand access to unhealthy commodities (food, alcohol, nicotine) and identify key characteristics of services | Desktop audit of apps and websites using a standardised data collection protocol | **Health**: Availability of unhealthy commodities on-demand  **Regulatory**: Legal aspects of access to age-restricted items  **Other**: Promotion strategies | - On-demand services for food, alcohol, and nicotine operate across urban and rural New Zealand - All services offered personal memberships and 97% used promotions - For services offering age restricted items, all had an age verification process; however, only 87% had birth date entry and 73% had an 18+ message pop-up on website entry - 60% of services appeared to have number limits on restricted items | - Desktop audit of publicly available information and policies; no physical audit of company behaviours - Access is not universal in all locations - Excluded direct food delivery by individual restaurants, potentially underestimating number of services available |

Abbreviations. GHG = greenhouse gas emissions; BMI = body mass index; PIQET = Packaging Impact Quick Evaluation Tool; SES = socioeconomic status; FES = Food Environment Score; PPE = personal protective equipment; HEI-2015 = Healthy Eating Index-2015; FFG = Five Food Group; PAEs = phthalic acid esters; HDDS = Household Dietary Diversity Scores; CO_2_ = carbon dioxide; NSW = New South Wales; ED = emergency department; OECD = Organisation for Economic Co-operation and Development
